# Supplementary material for: A lagrange programming neural network approach for nuclear norm optimization
Source: PLoS One. 2024 Feb 8;19(2):e0292380. doi: 10.1371/journal.pone.0292380 (PMC10852323; doi:10.1371/journal.pone.0292380)
Supplement: Attachment — Submitted filename: Response to Reviewers.docx [file pone.0292380.s001.docx]

Responses to the Comments and Suggestions of the Reviewers
(Manuscript ID PONE-D-23-12057)

**“A lagrange programming neural network approach for nuclear norm optimization”**

The authors are grateful to the reviewers and the Associate Editor for their efforts in reviewing the paper. In light of the insightful comments and constructive suggestions, the paper has been revised wherever possible. The point-to-point responses to the reviewers’ comments/suggestions are listed below, and revised parts in the manuscript are highlighted in blue.

**Responses to the Reviewer 1**

| Comments to the Authors | Authors’ Responses |
| --- | --- |
| 1.This article contains some publishable and modest results, but it is written badly. The use of English is not acceptable. Many sentences are incomplete without correct use of English grammar. Mathematics is also poorly written. | We have polished the whole paper. Moreover, we revised the math equations. |
| 2.The authors before Section 1.1 (Notations) introduce some notation (the nuclear norm). In that subsection, they introduced the Frobenius norm without calling it similarly to the spectral norm. | Some notations (i.e. the nuclear norm, et al.) are introduced before we use them. |
| 3.The inverse of a matrix is defined even for non-square matrices! I wonder where the adjoint will be used? | P is an operator, and we do not use it in the paper. Therefore, we delete the notation of P. |
| 4.Definition 1 is poorly stated, unclear. The same goes for Theorem 1. | We restated Definition 1 and Theorem 1. |
| 5.In the formulation of (10), why do you assume that the image and the preimages lie in the spaces of equal dimensions? | We assume that X is an m × n image, and h (X) is used as a constraint to calculate each element in X without changing the image dimension. |
| 6.Simulation study is fine to me. My recommendation to the authors is to re-write the manuscript paying attention to expressing Mathematical statements adequately and using the English language correctly. | We have polished the whole paper. Moreover, we revised the math equations. |

**Responses to the Reviewer 2**

| Comments to the Authors | Authors’ Responses |
| --- | --- |
| 1. In the introductory section, the introduction to LPNN does not flow naturally from the descriptions of the existing NNM approaches. Why did you have to use the LPNN for optimization? | We have elaborated on some of the advantages of LPNN in solving NNM. |
| 2. There seem to be a lot of typos/grammatical problems/unidiomatic expressions in this manuscript. It would be a good idea to edit the language carefully. | We have polished the whole paper. |
| 3. Why did you focus on solving NNM? NNM has been shown less effective in the context of low-rank matrix completion. The low-rank property of a certain matrix, actually, can be better characterized by the weighted/truncated nuclear norm, Schatten p-norm, direct exploitation of the discrete rank constraint in a projection manner, or preferably matrix factorization. You could still discuss the KKT convergence in these nonconvex cases. | Our main focus is to theoretically discuss the feasibility of using neural networks to solve NNM. We put forward some mathematical derivations about KKT convergence in nonconvex cases. |
| 4. Apart from the SVT, you should consider comparing your method to other off-the-shelf low-rank matrix completion schemes (e.g., those that I mentioned in my last concern). | We discussed other off-the-shelf low-rank matrix completion schemes in the introduction. |
