# Supplementary material for: A lagrange programming neural network approach for nuclear norm optimization
Source: PLoS One. 2024 Feb 8;19(2):e0292380. doi: 10.1371/journal.pone.0292380 (PMC10852323; doi:10.1371/journal.pone.0292380)
Supplement: Attachment — Submitted filename: Response to Reviewers.docx [file pone.0292380.s002.docx]

Responses to the Comments and Suggestions of the Reviewers
(Manuscript ID PONE-D-23-12057)

**“A lagrange programming neural network approach for nuclear norm optimization”**

The authors are grateful to the reviewers and the Associate Editor for their efforts in reviewing the paper. In light of the insightful comments and constructive suggestions, the paper has been revised wherever possible. The point-to-point responses to the reviewers’ comments/suggestions are listed below, and revised parts in the manuscript are highlighted in blue.

**First response**

**Responses to the Reviewer 1**

| Comments to the Authors | Authors’ Responses |
| --- | --- |
| 1.This article contains some publishable and modest results, but it is written badly. The use of English is not acceptable. Many sentences are incomplete without correct use of English grammar. Mathematics is also poorly written. | We have polished the whole paper. Moreover, we revised the math equations. |
| 2.The authors before Section 1.1 (Notations) introduce some notation (the nuclear norm). In that subsection, they introduced the Frobenius norm without calling it similarly to the spectral norm. | Some notations (i.e. the nuclear norm, et al.) are introduced before we use them. |
| 3.The inverse of a matrix is defined even for non-square matrices! I wonder where the adjoint will be used? | P is an operator, and we do not use it in the paper. Therefore, we delete the notation of P. |
| 4.Definition 1 is poorly stated, unclear. The same goes for Theorem 1. | We restated Definition 1 and Theorem 1. |
| 5.In the formulation of (10), why do you assume that the image and the preimages lie in the spaces of equal dimensions? | We assume that X is an m × n image, and h (X) is used as a constraint to calculate each element in X without changing the image dimension. |
| 6.Simulation study is fine to me. My recommendation to the authors is to re-write the manuscript paying attention to expressing Mathematical statements adequately and using the English language correctly. | We have polished the whole paper. Moreover, we revised the math equations. |

**Responses to the Reviewer 2**

| Comments to the Authors | Authors’ Responses |
| --- | --- |
| 1. In the introductory section, the introduction to LPNN does not flow naturally from the descriptions of the existing NNM approaches. Why did you have to use the LPNN for optimization? | We have elaborated on some of the advantages of LPNN in solving NNM. |
| 2. There seem to be a lot of typos/grammatical problems/unidiomatic expressions in this manuscript. It would be a good idea to edit the language carefully. | We have polished the whole paper. |
| 3. Why did you focus on solving NNM? NNM has been shown less effective in the context of low-rank matrix completion. The low-rank property of a certain matrix, actually, can be better characterized by the weighted/truncated nuclear norm, Schatten p-norm, direct exploitation of the discrete rank constraint in a projection manner, or preferably matrix factorization. You could still discuss the KKT convergence in these nonconvex cases. | Our main focus is to theoretically discuss the feasibility of using neural networks to solve NNM. We put forward some mathematical derivations about KKT convergence in nonconvex cases. |
| 4. Apart from the SVT, you should consider comparing your method to other off-the-shelf low-rank matrix completion schemes (e.g., those that I mentioned in my last concern). | We discussed other off-the-shelf low-rank matrix completion schemes in the introduction. |

**Second response**

**Responses to the Reviewer 2**

| Comments to the Authors | Authors’ Responses |
| --- | --- |
| The related works I suggested to include in their Introduction, namely [11-13], are not directly relevant to the nuclear norm minimization (NNM) problem (3). I request the authors to remove the incongruous citations from the sentence "many optimization approaches [5-13] were proposed to solve problem (3)" and reintroduce such works in the paragraph below, which begins with the sentence "Neural networks were used to address nonlinear equality constraint problems." | We removed inappropriate references and reintroduced them in the introduction. |
| It would be more appropriate to use the past tense or present perfect tense in the conclusion. | We modified the tense in the conclusion. |
| The authors should compare their LPNN-based method with other NNM schemes, in addition to the SVT technique. Examples of such schemes include fixed-point continuation [a] and ADMM [b]. | We compared the proposed algorithm (called LPNN) with ADMM and SVT in terms of image recovery. |

**Responses to the Reviewer 3**

| Comments to the Authors | Authors’ Responses |
| --- | --- |
| The contribution of the authors is not very clear, it must be highlighted in the abstract and the conclusion part explicitly. | We refined the contribution in the introduction. Moreover, we highlighted it in the abstract and the conclusion part. |
| The proof of Lemma 1 is not clear. How do equations (5a) and (5b) lead to equation (6)? | Based on the properties of the convex function, we added the two formulas and multiply the two sides by minus one. |
| The equivalence between (1) and (3) is not straightforawrd. Authors state that it is commonly used without giving any references. | The nuclear norm is convex, and the nuclear norm of the matrix is equivalent to the rank of the matrix. Therefore, we reformulated problem (1) to problem (3). |
| Authors use the term "best solution" in Proposition 1 without defining what is a "best solution". | The nuclear norm problem is a convex problem, and there must be an optimal solution. Hence, we assume that its optimal solution is X*, and we change "best solution" to the "optimal solution". |
| Authors must highlight that the nuclear norm is convex. | We highlighted that the nuclear norm is convex. |
| The convergence of the neural network is not studied. | We studied the convergence of the experiment in Section 3.1. Figures 2 and 3 respectively presented the optimal solution versa the convergence time. |
| The experimental Section is very bried and lacks information:  - What are the software used to implement the suggested neural network?  - The CPU time of the different methods is not mentionned.  - Authors claim that NNM-LPNN outperfoms SVT apporach without giving a true discussion leading to such conclusion. | We conducted experiments in MATLAB.  Since different algorithms do not build neural networks in the same way, and the algorithm has high optimization space in Time complexity, we only verified the feasibility of LPNN and showed the Rate of convergence of LPNN. The CPU time of code does not have a reference value, so we did not show the CPU time of code.  We have added more experiments and demonstrated through indicators that LPNN outperforms other algorithms of matrix completion in the experiment. |
| The quality of the plots is poor. | The uploaded figures were required to compress. The uncompressed figures were uploaded in the supporting information package. |
